# A Deep Learning Approach to Automatically Classify Ice Hockey Shooting Actions Using Acceleration Signals

**DOI:** 10.3390/s26113361

**Published:** 2026-05-26

**Authors:** Samuel Tremblay, Philippe J. Renaud, Shawn M. Robbins, David J. Pearsall, Philippe C. Dixon

**Affiliations:** 1 Department of Kinesiology & Physical Education, McGill University, Montreal, QC H2W 1S4, Canada; s.tremblay@lboro.ac.uk (S.T.); philjrenaud@hotmail.com (P.J.R.); david.pearsall@mcgill.ca (D.J.P.); 2 Centre for Interdisciplinary Research in Rehabilitation, Lethbridge-Layton-MacKay Rehabilitation Centre, Montreal, QC H4B 1T3, Canada; shawn.robbins@mcgill.ca; 3 School of Physical and Occupational Therapy, McGill University, Montreal, QC H3G 1Y5, Canada

**Keywords:** wearable sensors, human activity recognition, machine learning, ice hockey, shooting

## Abstract

**Highlights:**

**What are the main findings?**

- A convolutional neural network model using acceleration data accurately classified seven ice hockey-related actions.
- The average F1 score was 95.0% ± 3.0 using all sensors and 93.5% ± 1.6 using hand sensors.

**What are the implications of the main findings?**

- Machine learning approaches with minimal sensors can automatically classify ice hockey shooting actions.
- These methods can enhance training practices by providing objective performance metrics and allowing coaches to deliver data-driven feedback to players.

**Abstract:**

In ice hockey, automatic activity detection using wearable sensors and machine learning could provide objective feedback to support coaches and players during performance evaluation. The primary objective was to assess the predictive ability of a deep learning model to recognize common ice hockey stick striking actions (passing, shooting) from inertial measurement unit sensors. This study implemented a fully connected convolutional neural network model to classify seven ice hockey-related technical actions (wrist shot, slap shot, backhand shot, one-timers, pass, other, and rest) using acceleration data via two setups: an all-sensor configuration (17 sensors) and a hands-only sensor configuration (2 sensors) in 43 elite players. Data were split into 80/20 train/test sets, with a five-fold cross-validation applied to the training data. The train/test split was repeated 10 times with different random splits to assess stability of results. The model achieved high classification accuracy, with the all-sensor model reaching an average F1 score of 95.0 ± 3.0% and the hands-only model achieving 93.5 ± 1.6%. These findings support the use of convolutional neural networks for automatic shooting action classification in ice hockey and highlight the feasibility of using minimal sensor configurations, such as sensor-integrated gloves, for real-world applications. This approach could further enhance training practices by providing objective performance metrics and allowing coaches to deliver data-driven feedback to players.

## 1. Introduction

Performance analysis in sports has evolved from manual and subjective classification of game specific events to automatic recognition of sport specific movements by way of body worn sensors such as accelerometers and inertial measurement units (IMUs) [1]. Such wireless sensors now make it feasible to collect performance data and identify sport actions in challenging environments. Currently, ice hockey analytics and technical action recognition often use video-based approaches and local positioning systems [1]; however, in a high-speed team sport such as ice hockey, the environment, equipment worn, and rapid movements are challenges to collecting reliable kinematic measures. Adopting a machine learning analysis from wearable sensor data inputs may be an efficient way to automatically identify hockey actions.

Machine learning models can automatically identify athletic movements from a continuous data signal [2]. Convolutional neural networks (CNNs) are used for time-series classification tasks due to their ability to extract relevant features from raw data and have achieved or improved upon the state-of-the-art for classification accuracy [3,4,5,6]. IMUs combine accelerometers, gyroscopes, and magnetometers and are increasingly used in sports to quantify movement [7,8].

There has been minimal research evaluating automatic technical action recognition in hockey using sensor-based classification and machine learning. One study from Hardegger et al. [9] used a stick instrumented with an IMU, four strain gauges, and two pressure-sensitive potentiometers to differentiate between wrist and slap shots with 100% accuracy using a rule-based classifier in 19 male players completing 100 shots in total. The algorithm used stick orientation data before identifying stick deflection peaks to decide between shot types. The perfect accuracy reflects the different movement profiles of the stick and body during wrist and slap shots; however, other actions with similar profiles (one timer vs. slap shots) may confuse models. In a subsequent experiment, they used a random forest classifier to detect gameplay actions (wrist shots, slap shots, hits, turning breaking) in four male players using IMUs on both gloves and chest protector, yielding an overall F1 score of 0.7. Shooting and hitting actions had the highest accuracy because they involved distinct arm movements (wrist and slap shots) or involved sudden deceleration of the chest (hits). Turning actions had the lowest accuracy likely due to their limited arm motion. Despite sample size limitations, their work highlights the potential of IMU-based system for automated action recognition in hockey.

Although ice hockey action classification research remains limited, related studies in other sports have shown promising results. For example, machine learning models have classified action types in water polo [10], field hockey [11], golf [12], tennis [13], and volleyball [14] using various sensor configurations and models. These studies report classification accuracies ranging from 83.2% to 96.7%, further demonstrating the potential of wearable sensors and deep learning for analyzing dynamic athletic movements. Machine learning approaches using sensor data have also been utilized successfully to classify physical activities and activities of daily living in healthy adults and patient populations [15,16,17,18].

Despite promising classification results across studies, there is no clear consensus on optimal machine learning model architectures or implementation processes for recognizing striking motion actions. Variability in sensor configurations and limited transparency around model preprocessing, tuning, and evaluations make replication difficult [5,11,12,14,19]. Developing and standardizing models used in automatic recognition of ice hockey movements/actions would advance the science of this sport and benefit players, coaches and equipment manufacturers. It would support coaches and players by improving the efficiency of performance evaluation in practice and game scenarios. For instance, players and coaches could receive feedback on how often players are completing specific technical actions to ensure they perform adequate repetitions for learning, understand what players are doing during game situations giving insight into player tendencies, and provide measures of training load and rest. Equipment manufacturers could obtain measures of loading frequency, which may relate to equipment failure (e.g., stick breaking). Differentiating ice hockey actions is a foundational step towards a comprehensive, data-driven analysis of execution quality, skill level, and player development. Therefore, the study aimed to evaluate the performance of a CNN deep learning model to automatically classify distinct ice hockey technical actions, specifically stick striking (e.g., shooting, passing), performed by elite hockey players using three-dimensional acceleration data from a commercial IMU system. The secondary aim was to determine the minimum sensor configuration subset of the 17-sensor system. It was hypothesized that the CNN model would be able to automatically identify the different technical actions and that an all-sensor configuration (17 sensors) would show the strongest performance.

## 2. Materials and Methods

### 2.1. Participants

Forty-three (27 males, 16 females) elite ice hockey players of both shot handedness (left = 29, right = 14) (Table 1) participated in this cross-sectional study. Participants were recruited from McGill University’s varsity teams and the broader Montreal hockey community. “Elite” was defined as athletes with experience in leagues such as the Canadian Hockey League (Junior), USports (University), or equivalent. All participants were skaters (non-goaltenders), injury-free, and provided written informed consent prior to participation. The study was approved by the McGill University Research Ethics Board II in accordance with the Tri-Council Policy Statement on Ethical Conduct for Research Involving Humans (REB#375-0216).

### 2.2. Data Collection

**Figure 1:**
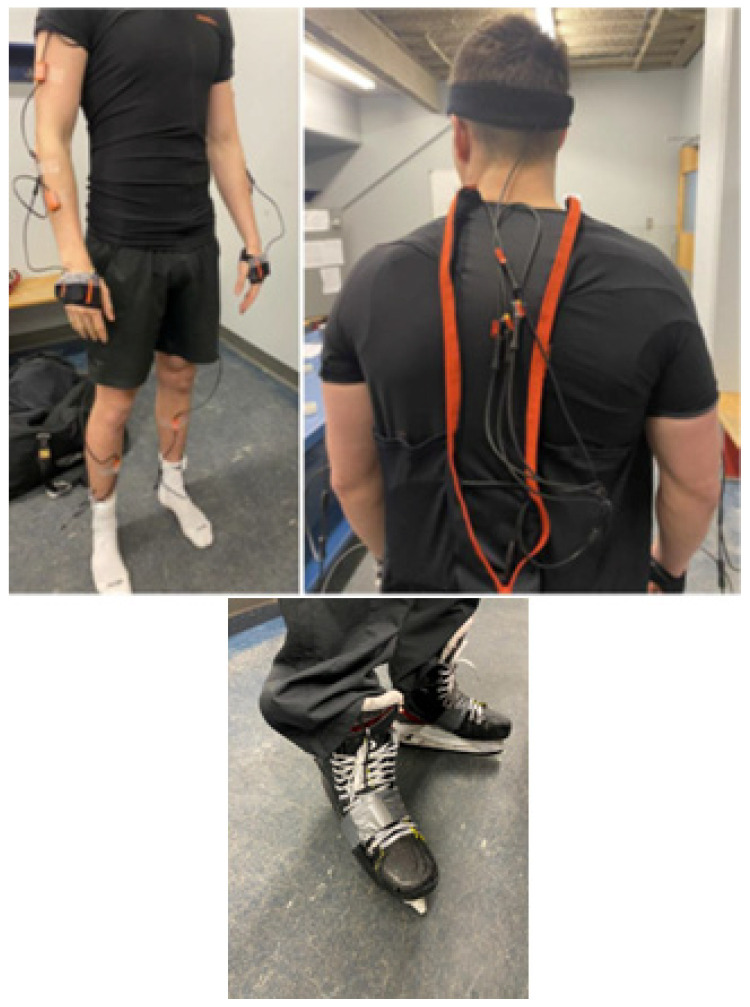
Sensor and wire placement on participant and skates.

This study was conducted on an indoor ice surface (McConnell arena, Montreal, QC, Canada) from September to December 2021. Participants’ demographics (e.g., age) and anthropometrics were recorded. Movement data were collected using the Xsens MVN Link system (Movella, Enschende, The Netherlands), consisting of 17 IMUs positioned on the head, sternum, pelvis, upper arms, forearms, thighs, lower legs, and feet, connected through wires and consistent with previous guidelines (Figure 1; [20]). The sensors were placed on the body with double-sided tape and secured with medical tape, except for the head (in a headband), hands (inside gloves), and feet (taped on skate laces). Sensor orientations were consistent with manufacturers’ guidelines [21] resulting in a coordinate system of Y as vertical, Z as medial–lateral, and X as anterior–posterior. Players wore their own gloves, skates, and sticks, but did not wear full protective equipment to avoid interference with sensor attachments. Sensors recorded 3D accelerations (range ±160 m/s^2^) at 240 Hz and transmitted the data wirelessly through a Wi-Fi access point. The system was calibrated for each participant using manufacturer guidelines, including a static pose and walking forward in a straight line [21]. These IMUs have proved to be accurate and reliable when measuring kinematics during other sporting tasks [22,23].

A Bonita video camera (Vicon Motion Systems Ltd., Oxford, UK) recorded visual footage at 30 Hz. It was synchronized with the Xsens system using the Xsens SyncStation (Movella, Enschende, The Netherlands), allowing simultaneous recording for action labeling during preprocessing.

**Figure 2:**
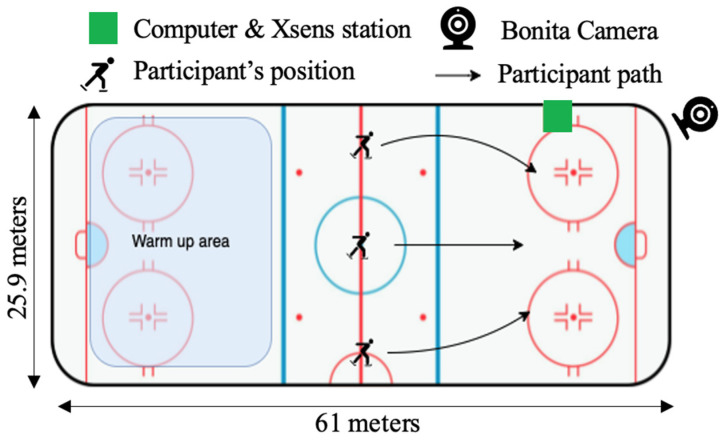
Top view of the on-ice data collection layout for wrist and slap shots. Each technical action was performed from three red line starting positions, with five trials per position (15 total per shot type). Participants skated towards the net and initiated the shot after crossing the top of the face-off circle.

Following a 5 min on-ice warm-up, testing was performed during a 30 min session consisting of seven ice hockey technical actions. The technical actions performed in a randomized order included 15 wrist shots (WS), 15 slap shots (SS), 10 backhand shots (BH), 10 one-timers (OT), 5 passes, 5 stick-handling trials, and a 5 min resting period. We were interested in classifying stick striking actions (i.e., shooting, passing), while other actions (stick-handling, rest) were included to potentially confuse the model and replicate typical game-like actions. A 30 s rest was allowed between trials. The on-ice layout was consistent across all participants. For wrist and slap shots, players started from three predefined positions along the red line, completing five trials from each location. They skated toward the net and executed the shot after crossing the face-off circle (Figure 2). Backhand shots were performed from a single starting point aligned with the player’s backhand side (e.g., left side for right-handed shooters). Players skated towards the net and executed the shot. For the one-timer shots, players remained stationary near the face-off dot (e.g., left side for right-handed players) and received passes from the study’s principal investigator, an elite-level player, positioned at the opposite face-off dot. During the passing action, players skated from the second blue line and passed to the principal investigator, who stood opposite to their handedness at the far goal line, after crossing the first blue line. For stick-handling, players skated from one blue line to the other while handling the puck using the technique they would use in a game. Detailed diagrams of each technical action layout are presented in Supplementary Materials (Figures S1–S4). Players were only instructed to perform the action but were not provided additional instructions on how to perform the action to ensure they were performing them naturally and there would be variability between players.

### 2.3. Data Preprocessing

Only the gravity-removed acceleration data were selected for the analysis to create the simplest model that was computationally efficient and allow future research to use less expensive sensors. Acceleration-based signals are also less susceptible to drift accumulation errors than gyroscope orientation or position estimates, making them well suited for short-duration movement classification tasks. Additionally, a previous study demonstrated that acceleration signals from optical motion capture systems were able to accurately classify ice hockey actions [24]. Four participants’ datasets were excluded due to sensor detachment or file corruption, leaving 39 clean datasets. The deep learning model preprocessing and its implementation were done using Python software (version 3.12.13, Python Software Foundation, https://www.python.org/) on Google’s Collaboratory Pro+ GPU (GPU: 1xTesla P100, 54.8 GB RAM). The code is available at https://github.com/SamTremblay18/Hockey-Shot-Classification (accessed on 2 March 2026).

Action segmentation was performed manually using the synchronized video footage. One investigator identified when the motion for each technical action began and ended at the completion of follow-through. For each trial, three labels were assigned: “Pre” (motion preceding the action), the corresponding action label (e.g., WS, SS, OT, BH, pass), and “Post” (follow-through until start of next trial). These were based on video timestamps, upsampled to match the IMUs’ 240 Hz sampling rate. Given the 240 Hz frame rate, temporal boundaries could be localized within a small number of frames (on the order of a few milliseconds), although some subjectivity remains in defining transitional phases (“Pre” and “Post”). Two sensor configurations were created: all-sensor configuration, which used all 17 IMUs (17 × 3 axes = 51 channels), and hands-only sensor configuration, using only the two hand sensors (2 × 3 axes = 6 channels). The hand-only sensor configuration was selected since there are fewer sensors, which is more practical, and it would be more easily implemented in future applications. The hand sensors were selected since the actions (e.g., WS, pass, etc.) require quick and/or large amplitude arm movements compared to other hockey related skills (e.g., skating). During the “Pre”, “Post” phases, players were typically stick-handling, carrying the puck, or skating to pick up a puck. Since they had similar movement patterns and were distinct from stick striking actions, “Pre” and “Post” and stick-handling trials were grouped under a unified label “other”. These data were included to potentially confuse the models in identifying the stick striking actions of interest (WS, SS, OT, BH, pass) as players can move this stick quickly while stick-handling. This resulted in seven final labels: WS, SS, OT, BH, pass, “other” and rest. All stick-related trials (WS, SS, BH, OT, pass) were standardized to a fixed input length of 576 frames (2.4 s; maximum observed trial length). Shorter trials were padded with frames from the preceding “other” segment, maintaining temporal continuity in the acceleration signal rather than zero-padding or resampling, which could distort the original signal. A Preliminary sensitivity analysis of padding technique showed no major differences in model performance between zero-padding and “frame padding”. This approach also allowed the inclusion of potential cues from the preparatory phase. To address class imbalance, only 15 “other” trials per participant were randomly selected. For the rest class, the first 15 segments of 576 frames during the seated bench period were used. This resulted in approximately 85 trials per participants, composed of 15 WS, 15 SS, 10 BH, 10 OT, 5 passes, 15 “other” (i.e., Pre, Post, stick-handling) and 15 rest trials. Next, the action labels were encoded to integers, as required for input to a deep neural network [25] and each participant’s data were formatted into a tensor of shape *n* × 576 × *ch*, where *n* is the number of trials (~85 per participants), and *ch* is the number of channels (51 for all-sensor and 6 for hands-only configurations). The encoded labels had their own one-dimension tensor with a shape corresponding to the participants’ trials. The next step involved a randomized inter-subject split of the 39 participants into distinct training, validation, and test groups. In total, 20% (n = 8) of participants were held out during model development as a test set to be used only for final model performance evaluation [25]. The remaining 80% (n = 31) were used to generate and store five folds for later use during the model development phase. Each fold consisted of a randomized split of participants into training (n = 27) and validation (n = 4) group. This inter-subject split approach prevented any data leakage to the test set, preserving independence during training and evaluation [25]. This procedure was repeated 10 times with different randomized splits to estimate variability of the models’ performance metrics. Finally, the training dataset (i.e., after the data split) was z-score normalized by subtracting the mean and dividing by the standard deviation, facilitating the model’s learning process by having each value centered around 0 with a standard deviation of 1 [25]. Also, we did not adjust for handedness (e.g., left vs. right shooter) to observe how the model would perform in its rawest form.

### 2.4. Deep Learning Model Architecture

The deep learning model developed in this study was based on the Fully Convolutional Network (FCN) architecture originally proposed by Wang, Yan, and Oates [23] for end-to-end time series classifications. FCN is a type of CNN, and these models have shown to be superior for classifying human activities compared with conventional machine learning approaches [8]. The automatic feature extraction from deep learning models is a distinct advantage compared to algorithms that rely on manual feature engineering from researchers that may miss important signal features [7]. Such deep learning networks are attractive because of their simplicity, versatility, and scalability [25]. CNNs are well equipped to handle time series data that measure motion and can learn from large data sets [7,8,9,10,11,12,13,14,15,16,17,18,19,20,21,22,23,24,25,26,27]. To reduce overfitting, dropout layers and kernel regularizers were added following Chollet’s recommendations [25]. This method was used to build a simple and reusable framework for future ice hockey classifying technical actions.

**Figure 3:**
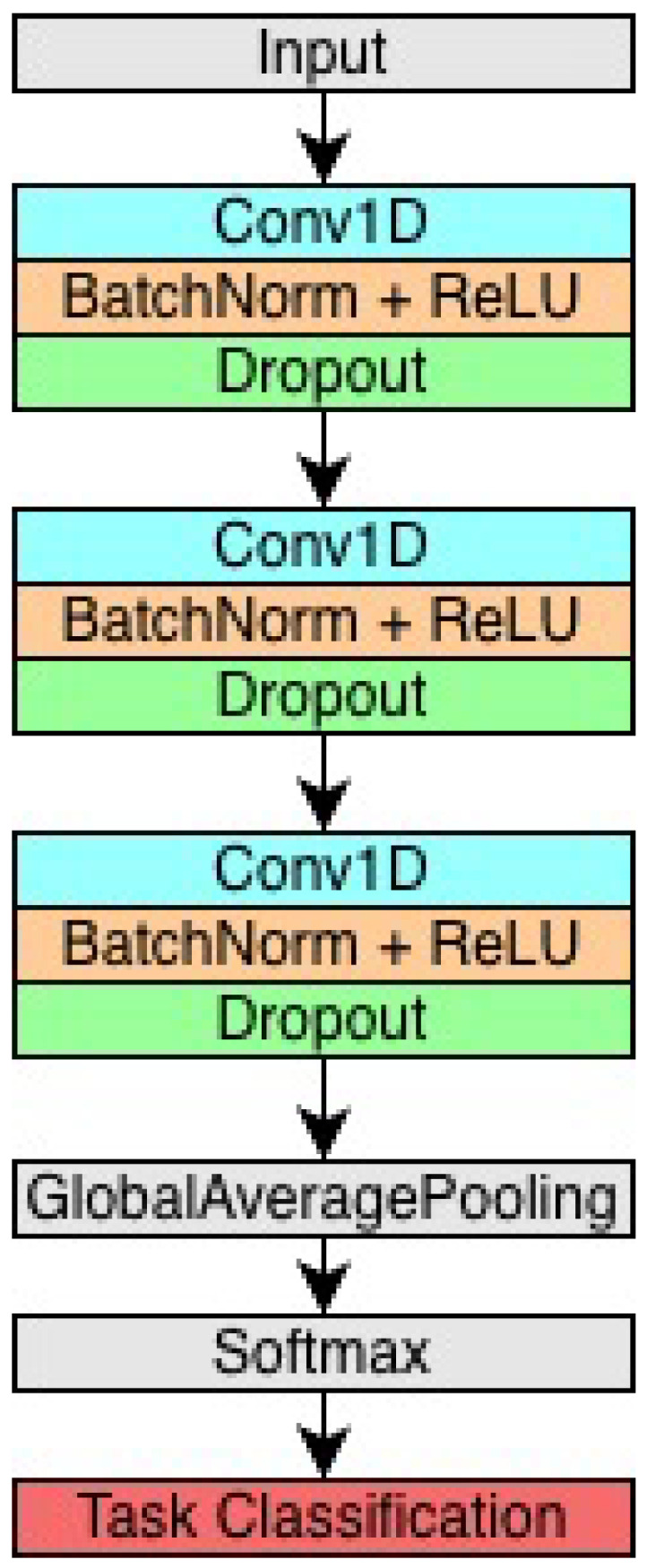
Schematic of the fully convolutional network architecture.

A baseline model, corresponding to the original FCN architecture without dropout layers, kernel regularization, and default hyperparameters, was first implemented as a reference for performance comparison. Each model (all-sensor and hands-only configuration) received input tensors of shape *n* × 576 × *ch*. The model architecture consisted of three convolutional blocks, each comprising a 1D convolutional layer, batch normalization, ReLU activation and dropout. Following their respective convolutional blocks, both architectures fed into a global average pooling layer instead of a fully connected layer, reducing the number of parameters and further limiting overfitting [26]. A final softmax layer outputted the class probabilities across seven technical actions: WS, SS, OT, BH, pass, rest, and “other” (Figure 3).

A 5-fold cross-validation was used to tune and evaluate hyperparameters through KerasTuner’s random search [28]. The tuner was executed separately for each sensor configuration and fold, and the best combination yielding the lowest validation loss was recorded. After all folds, hyperparameter configurations were aggregated into a final set in which numerical values were averaged and rounded, and categorical choices were selected by majority vote (Table 2). This final configuration was trained on the complete training data and evaluated on the held-out test set.

After tuning, the model was trained on all 31 training participants using the hyperparameters. The optimizer type (Adam, SGD, RMSprop) was also treated as a tunable hyperparameter. As a result, different optimizers were selected for both sensor configurations based on their respective validation performance for each random split. The sparse categorical cross entropy loss function was used, selecting sparse categorical accuracy to evaluate the model’s training. The learning rate, set at 1 × 10^−5^, was monitored by the validation loss and automatically reduced by a factor of 0.5 if validation loss did not improve through a callback with a patience of 6 epochs. The FCN models were trained for a maximum of 500 epochs with a batch size of 64. A Keras early stopping callback with a patience of 20 epochs was implemented to avoid overfitting.

### 2.5. Evaluation Metrics

Model performance was evaluated on the held-out test set (n = 8) using F1 scores, precision, recall, and specificity [29]. Precision reflects the proportion of correctly predicted technical actions among all predictions for a given class. Recall represents the proportion of actual actions being correctly predicted. F1 score can be interpreted as the weighted average of the precision and recall, providing a balanced measure of both. A high F1 score indicates strong performance in identifying and avoiding false predictions. Hence, this will be the primary metric of interest when comparing both sensor configurations. Finally, 7 × 7 confusion matrices displaying true and predicted labels across all actions for both sensor configurations were generated to visualize classification performance and potential misclassifications [29].

## 3. Results

Model performances were consistent across training, validation, and test sets, indicating no evidence of overfitting. During training on the first split, accuracy (all-sensor: training = 0.99, validation = 0.98; hands-only: training = 0.97, validation = 0.96) and loss values for the baseline model were closely aligned, and their curves showed no divergence. Model performance was initially benchmarked against this baseline FCN model, proposed by Wang, Yan, and Oates [26]. The introduction of dropout layers, kernel regularization, and hyperparameter optimization further mitigated overfitting risks and resulted in improved classification performance across both sensor configurations. When retrained using the optimized hyperparameters and evaluated on the independent test set for the first split, performance remained comparable (test accuracy: all-sensor = 0.98; hands-only = 0.95), suggesting the models generalized well. Finally, validation accuracy during 5-fold cross-validation was stable across folds (all-sensor = 0.97 ± 0.01; hands-only = 0.94 ± 0.02).

### 3.1. Model Performance for the All-Sensor Configuration

**Figure 4:**
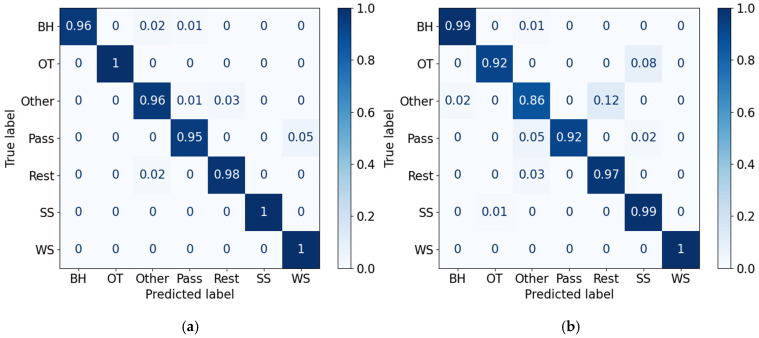
Representative confusion matrices for backhand (BH), one-timer (OT), “other”, pass, rest, slap shots (SS) and wrist shots (WS) of the FCN model for the (**a**) all-sensor configuration and (**b**) hands-only sensor configuration.

The FCN model had strong performances for the all-sensor configurations, demonstrated by high performances of F1, precision, recall, and specificity scores, with all scores ranging from 80.1% to 99.8% (Table 3). For all technical actions, the model revealed a mean average F1 score from the ten splits of 95.0 ± 3.0% compared to 94.1 ± 2.1% of the baseline model. The actions that generated the highest F1 score were the slap shot and one-timers with 98.1 ± 1.7% and 98.8 ± 2.4% respectively. Of note, the passing action had the lowest score overall with an 82.1 ± 13.9% F1 score. When looking at a representative confusion matrix, the “other” action (which included Pre, Post and stick-handling) had the most false positives and negative predictions and was mostly confused by the resting action (Table 3 and Figure 4a).

### 3.2. Model Performance for the Hands-Only Sensor Configuration

Overall, the hands-only configuration had strong performances with all scores ranging from 87.2 ± 2.5% to 99.5 ± 0.3% (Table 4). For all actions, the model averaged an F1 score of 93.5 ± 1.6% compared to 92.9 ± 2% of the baseline model. The action that generated the highest F1 score was the backhand shot with 96.9 ± 2.2%. For this sensor configuration, the pass action struggled the most with a mean F1 score 87.9 ± 6.6%. Looking at a representative confusion matrix (Figure 4b), the “other” action had the most false negative predictions, again being confused with the resting action. Also of note, the one-timer action had the second most false negative predictions, all against the slap shot action (Table 4 and Figure 4b).

## 4. Discussion

Although the automatic recognition of movements/actions using accelerometers/IMUs and machine learning has been examined in other sports, its application in ice hockey is novel. The current study advances previous work and includes 39 participants with over 3500 ice hockey technical actions. The FCN model successfully classified all seven technical actions, achieving a mean F1 score of 95.0 ± 3.0% for the all-sensor configuration and 93.5 ± 1.6% for the hands-only configuration. The hands-only configuration results highlight the feasibility of using sensors embedded in gloves to recognize hockey shooting related actions with high accuracy, providing manufacturers, coaches and players with objective, sensor-based performance feedback. The next step is to evaluate similar models on real game data.

Using only two sensors reduced the input signal size (51 all-sensor vs. 6 hands-only), reducing the amount of information the model could be trained on. Previous studies have shown that full body measures provide substantial information that could improve action identification. For example, in a 3D kinematic analysis study that did not involve machine learning, Michaud-Paquette et al. [30] found that lower limb and torso kinematics were predictors of wrist shot accuracy. Thus, training the hands-only configuration model without important information from the lower limbs and trunk may have contributed to its slightly lower average F1 score. Although the all-sensor configuration had greater data richness, it is not practical in games or practices. The hands-only configuration has greater usability and feasibility due to fewer IMUs, while still offering a high level of performance. Furthermore, not adjusting the models for shot handedness was not an issue considering the strong performance of all models. The models included acceleration data from both hands, resulting in mirror-image acceleration profiles for left- and right-handed shooters.

The hand-only configuration was accurate because most technical actions involved large or quick accelerations of the hand that were distinct from other actions. For instance, the classification of wrist shots, slap shots, and one-timers did not suffer from having only two sensors, achieving F1 scores of 94.7% to 95.7%. There were no misclassifications between wrist, slap, and backhand shots in the representative confusion matrix (Figure 4b). Slap shots involve full body wind-up and fast downswings, while wrist shots require rapid upper body movement for quicker releases. Backhands tend to be slower shots with the arms moving in a different direction than wrist and slap shots. These variations in shot techniques resulted in differing acceleration profiles and accurate classification. Other ice hockey actions that have less distinct hand motions would likely not perform well with the only-hand acceleration data, such as distinguishing between stopping and turning. Also, the study was conducted in a controlled environment and the high classification metrics should be interpreted with caution. It is likely that in-game data would be more difficult to classify due to greater variability in the execution of the technical actions.

Some technical actions were less distinguishable and resulted in errors. For instance, the model predicted slap shots when participants performed one-timers on occasion (Figure 4b). This was not unexpected, as many one-timers are performed, like a slap shot, producing similar hand accelerations between the two shot types. Pass classification was more challenging for both configurations, achieving 86.3% and 88.5% recall for all-sensor and hands-only configurations, respectively. Some passes were misclassified as wrist shots (Figure 4a) likely due to hard passes requiring quick movements of the hands and arms which would be similar to wrist shots. The variability in how passes are completed (slower, shorter passes vs. faster passes) will make it more difficult to classify. Finally, the “other” class presented the most misclassifications, being classified as other actions such as backhand, pass, or rest (Figure 4). This label grouped different movements such as stick-handling, skating transitions (Pre), and various post-shot behaviors (Post). Post-trial instructions were deliberately open-ended, leading to variability in how participants transitioned to the next trial. Some players casually skated or remained relatively still, which lead to misclassification as a rest action. Other players played with the puck leading to misclassification as backhand or passing. Lastly, the model performed almost perfectly in specificity scores for both sensor configurations. However, this should be interpreted with caution, as the multi-class structure results in a large number of true negatives, which can inflate specificity values. As such, precision, recall, and F1-score provide more informative insight into class-specific performance, particularly for more variable actions such as passes and “other”.

These findings support earlier work [10], which demonstrated the feasibility of machine learning and IMU-based classification in hockey. While the previous study included a variety of gameplay actions with a smaller sample size, the present work focused specifically on shooting action classification using a larger cohort of 39 elite players. A smaller dataset can reduce the predictive power and generalizability of machine learning models [31]. By using a larger and more focused dataset, this study aimed to enhance classification reliability and provide a more targeted contribution to hockey performance analysis.

This study provides a strong and feasible baseline method in ice hockey shooting-related action classification. The hands-only configuration offers a scalable, equipment-compatible solution for coaches and analysts to objectively assess performance, reducing reliance on subjective observation, cognitive bias, and time-intensive video review [8]. Practical examples could include sport scientists/coaches examining objective data (e.g., accelerations) during specific actions (e.g., shooting) and identifying which players do not match their peers and thus require further training. Hockey equipment manufacturers could examine the accelerations and frequency of stick loading, which could be used to develop fatigue models. Strength coaches and trainers could also use this data to monitor decreases in performance, which may be related to fatigue or injury. Future implementations should focus on embedding IMUs in gloves and pairing them with a sensor-instrumented stick (e.g., strain gauges) to assess shot mechanics in more detail and enable players to wear their full protective equipment. A live streaming classification pipeline could further enhance training environments, allowing athletes and coaches to receive immediate performance feedback.

This study had several limitations. The ecological validity was limited. Participants did not wear full equipment due to sensor placement constraints and data were collected in a controlled environment. This allowed the researchers to monitor sensor placement; however, future research should be tested with full equipment. Action types were also limited to isolate shooting and stick-handling actions; in-game transitions or skating techniques were not included. Other passing techniques (e.g., backhand passing) were also not included. Moreover, the present work focused on evaluating the performance of an end-to-end deep learning approach rather than establishing superiority over traditional feature-based models. Future research should include systematic comparisons with feature-based models to better understand the advantages of deep learning models in this application. The study included elite hockey players and results cannot be generalized to other groups such as youth players. The sample size, while stronger than previous studies, would still benefit from an increased number of participants. Future studies should examine different machine learning models, sensor configurations, and other signals (e.g., gyroscope) to determine if they improve accuracy and efficiency similar to other studies classifying everyday activities [16,17].

## 5. Conclusions

The FCN model proposed accurately classified seven on-ice hockey technical actions using both full-body and hand-only configurations. While the all-sensor configuration achieved the highest F1 score (95.0%), the hands-only configuration performed nearly as well (93.5%). Thus, the hands’ 3D acceleration data alone becomes a practical option in terms of tracking player shooting skill execution in training and game contexts. Future research should explore a greater variety of hockey technical actions (e.g., saucer and backhand passes) by including more game-like scenarios, more skating actions, different types of players, and exploring more machine learning approaches. Examining which inputs were most effective at action classification requires further exploration and may assist in simplifying the model. Adding player characteristics (e.g., age, competition level, position) may also improve the accuracy of action classification if more heterogenous player samples are studied. Ultimately, these approaches should be evaluated in-game with real-time deployment to establish ecological validity.

## Figures and Tables

**Table 1:** Descriptive statistics of participants.

	Age (Yrs) Mean ± SD	Height (cm) Mean ± SD	Mass (kg) Mean ± SD	Experience (Yrs) Mean ± SD
Men (n = 27)	28 ± 2	182.7 ± 6.0	84.0 ± 7.7	18 ± 3
Women (n = 16)	25 ± 2	168.5 ± 4.4	67.3 ± 7.1	14 ± 3
All (n = 43)	27 ± 3	177.4 ± 8.8	77.8 ± 11.0	17 ± 3

**Table 2:** Selected hyperparameters for the final tuned models. Values in brackets correspond to the three convolutional layers.

Hyperparameter	Search Range/Options	Step Size
Filter size	64 to 256	64
Kernel size	3 to 5	1
Regularizer	‘None’, ‘L1’, ‘L2’	–
Dropout	0 to 0.5	0.1
Optimizer	‘Adam’, ‘SGD’, ‘RMSprop’	–

**Table 3:** Mean (±standard deviation) model performance in % for the All-Sensor Configuration on the test set. The highest scores for each metric are bolded.

Technical Actions	Precision	Recall	F1 Score	Specificity
Backhand	95.8 ± 4.2	96.3 ± 4.8	95.9 ± 3.6	99.4 ± 0.6
One-timer	98.3 ± 4.2	**99.4 ± 0.9**	**98.8 ± 2.4**	99.8 ± 0.6
Other	94.1 ± 3.2	92.4 ± 3.4	93.2 ± 2.5	98.8 ± 0.7
Pass	80.1 ± 19.0	86.3 ± 12.2	82.1 ± 13.9	98.3 ± 1.9
Rest	95.1 ± 2.7	97.5 ± 2.7	96.3 ± 1.6	98.9 ± 0.6
Slap shot	**99.0 ± 2.2**	97.3 ± 3.1	98.1 ± 1.7	**99.8 ± 0.5**
Wrist shot	96.2 ± 3.4	91.6 ± 10.9	93.6 ± 6.9	99.2 ± 0.7
Mean Weighted average	95.4 ± 2.6	95.0 ± 3.0	95.0 ± 3.0	-

**Table 4:** Mean (±standard deviation) model performance in % for the Hands-Only Sensor Configuration on the test set. The highest scores for each metric are bolded.

Technical Action	Precision	Recall	F1 Score	Specificity
Backhand	96.5 ± 2.6	**97.4 ± 2.9**	**96.9 ± 2.2**	99.5 ± 0.4
One-timer	95.1 ± 3.8	95.8 ± 3.4	95.4 ± 1.9	**99.5 ± 0.3**
Other	91.4 ± 3.6	87.2 ± 2.5	89.2 ± 2.1	98.3 ± 1.0
Pass	88.3 ± 7.9	88.5 ± 10.9	87.9 ± 6.6	99.5 ± 0.5
Rest	90.8 ± 1.7	94.7 ± 2.9	92.7 ± 1.5	97.8 ± 0.5
Slap shot	**97.2 ± 1.9**	92.5 ± 5.5	94.7 ± 2.5	99.4 ± 0.4
Wrist shot	94.4 ± 5.2	97.2 ± 2.6	95.7 ± 2.7	98.7 ± 1.4
Mean Weighted average	93.7 ± 1.5	93.5 ± 1.6	93.5 ± 1.6	-

## Data Availability

The datasets presented in this article are not readily available because consent was not obtained from the research participants. The machine learning code is available at https://github.com/SamTremblay18/Hockey-Shot-Classification, accessed on 14 March 2026.

## Supplementary Materials

The following supporting information can be downloaded at: https://www.mdpi.com/article/10.3390/s26113361/s1, Figure S1: Top view, on-ice data collection layout for backhand shots (right-handed participants); Figure S2: Top view, on-ice data collection layout for one-timer shots (right-handed participants); Figure S3: Top view, on-ice data collection layout for the passing action (right-handed participants); Figure S4: Top view, on-ice data collection layout for stick-handling action (right-handed participants).
