# Supplementary material for: A Deep Learning Approach to Automatically Classify Ice Hockey Shooting Actions Using Acceleration Signals
Source: Sensors (Basel). 2026 May 26;26(11):3361. doi: 10.3390/s26113361 (PMC13259433; doi:10.3390/s26113361)
Supplement: Supplementary file 1 [file sensors-26-03361-s001.zip › sensors-4229970-supplementary.pdf]

Supplementary Materials

A Deep Learning Approach to Automatically Classify Ice Hockey Shooting Actions Using Acceleration Signals

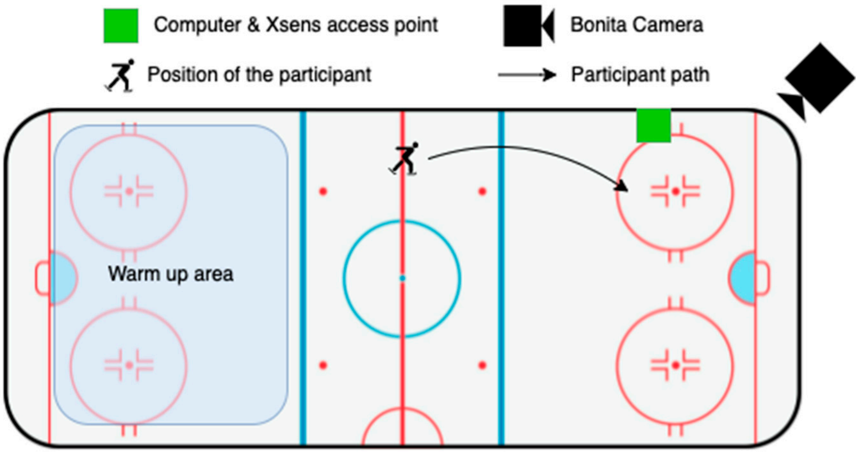

Figure S1. Top view, on-ice data collection layout for backhand shots (right-handed participants). Participants were positioned at a single location on their backhand side. This means that a right-handed participant was positioned on the left side of the ice, and a left-handed shooter was positioned on the right side of the ice. Participants skated towards the net and started their shooting motion after crossing the top of the circle area. A total of 10 backhand shots were performed by each participant.

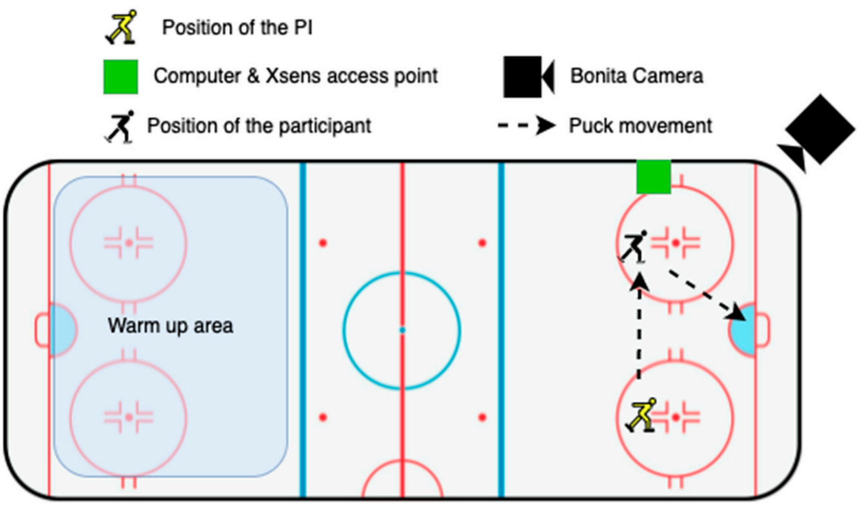

Figure S2. Top view, on-ice data collection layout for one-timer shots (right-handed participants). The principal investigator (PI) of this study was the puck passer and also considered as an elite hockey player. Both the participant and PI remained still between the face-off dot and the top of the circle on their respective side of the ice. Right-handed participants were positioned on the left side of the net while left-handed participants were positioned on the right side of the net. Each trial consisted of a pass of the puck going from the PI to the participant, so that they could in turn perform a one-timer. A total of 10 one-timer shots were performed by each participant.

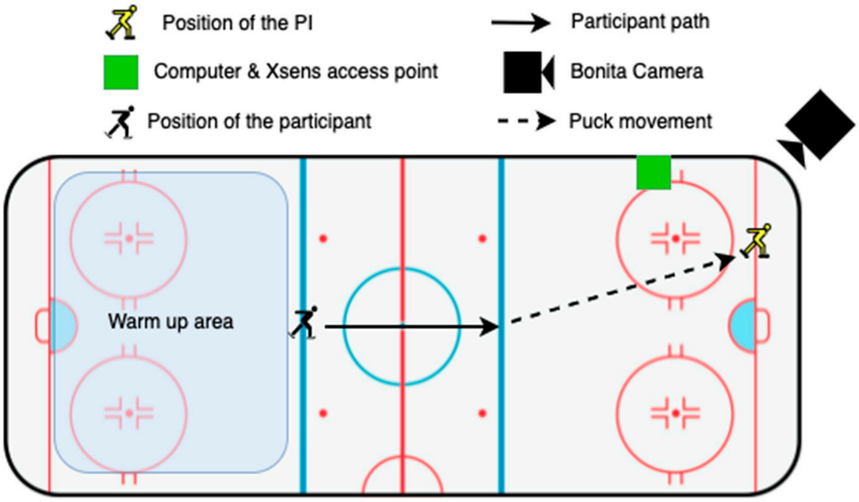

Figure S3. Top view, on-ice data collection layout for the passing action (right-handed participants). Participants were positioned at the second blue line and the PI was positioned on the goal line on the opposite side of the participant's handedness. Thus, for a right-handed participant, the PI was positioned on the left side of the net and for a left-handed participant, the PI was positioned on the right side of the net. For each trial, the participant skated forward with the puck and performed a pass to the PI when reaching the first blue line. A total of 5 trials were recorded.

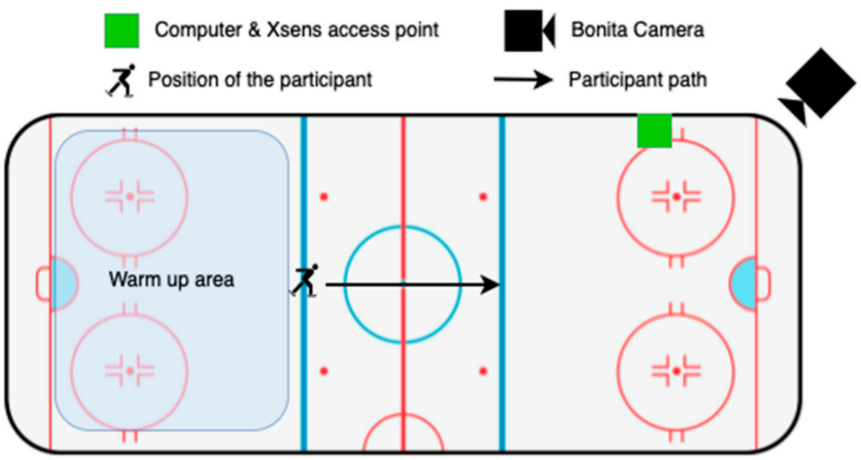

Figure S4. Top view, on-ice data collection layout for stick-handling action (right-handed participants). Participants started in the same position as in the passing action. Participant were asked to skate forward and stick-handle the puck at the same time either on the forehand or in front (however they felt comfortable), replicating a similar stick-handling pattern as in the start of a wrist or a slap shot trial. Participants skated while stick-handling all the way to the next blue line. A total of 5 trials were recorded.
